# Gold nanoparticles attenuate the interferon-γ induced SOCS1 expression and activation of NF-κB p65/50 activity via modulation of microRNA-155-5p in triple-negative breast cancer cells

**DOI:** 10.3389/fimmu.2023.1228458

**Published:** 2023-08-31

**Authors:** Aisha Farhana, Abdullah Alsrhani, Naila Rasheed, Zafar Rasheed

**Affiliations:** ^1^ Department of Clinical Laboratory Sciences, College of Applied Medical Sciences, Jouf University, Sakaka, Aljouf, Saudi Arabia; ^2^ Department of Medical Biochemistry, College of Medicine, Qassim University, Buraidah, Saudi Arabia; ^3^ Consultant, Calamvale, QLD, Australia; ^4^ Department of Pathology, College of Medicine, Qassim University, Buraidah, Saudi Arabia

**Keywords:** gold nanoparticles, SOCS1, interferon-γ, hsa-miR155-5p, NF-κB, triple negative breast cancer cells, nanomodulation

## Abstract

**Objective:**

Triple-negative breast cancer (TNBC) is a very aggressive form of cancer that grows and spreads very fast and generally relapses. Therapeutic options of TNBC are limited and still need to be explored completely. Gold nanoparticles conjugated with citrate (citrate-AuNPs) are reported to have anticancer potential; however, their role in regulating microRNAs (miRNAs) in TNBC has never been investigated. This study investigated the potential of citrate-AuNPs against tumorigenic inflammation via modulation of miRNAs in TNBC cells.

**Methods:**

Gold nanoparticles were chemically synthesized using the trisodium-citrate method and were characterized by UV-Vis spectrophotometry and dynamic light scattering studies. Targetscan bioinformatics was used to analyze miRNA target genes. Levels of miRNA and mRNA were quantified using TaqMan assays. The pairing of miRNA in 3'untranslated region (3'UTR) of mRNA was validated by luciferase reporter clone, containing the entire 3'UTR of mRNA, and findings were further re-validated via transfection with miRNA inhibitors.

**Results:**

Newly synthesized citrate-AuNPs were highly stable, with a mean size was 28.3 nm. The data determined that hsa-miR155-5p is a direct regulator of SOCS1 (suppressor-of-cytokine-signaling) expression and citrate-AuNPs inhibits SOCS1 mRNA/protein expression via modulating hsa-miR155-5p expression. Transfection of TNBC MDA-MB-231 cells with anti-miR155-5p markedly increased SOCS1 expression (p<0.001), while citrate-AuNPs treatment significantly inhibited anti-miR155-5p transfection-induced SOCS1 expression (p<0.05). These findings were validated by IFN-γ-stimulated MDA-MB-231 cells. Moreover, the data also determined that citrate-AuNPs also inhibit IFN-γ-induced NF-κB p65/p50 activation in MDA-MB-231 cells transfected with anti-hsa-miR155-5p.

**Conclusion:**

Newly generated citrate-AuNPs were stable and non-toxic to TNBC cells. Citrate-AuNPs inhibit IFN-γ-induced SOCS1 mRNA/protein expression and deactivate NF-κB p65/50 activity via negative regulation of hsa-miR155-5p. These novel pharmacological actions of citrate-AuNPs on IFN-γ-stimulated TNBC cells provide insights that AuNPs inhibit IFN-γ induced inflammation in TNBC cells by modulating the expression of microRNAs.

## Introduction

Breast cancer presents as the most common cancer pathology in women, other than non-melanoma skin cancer, all over the globe (1). At the molecular denomination, breast cancer has now been understood as a heterogeneous disorder. Its current treatment is curable in 70-80% of patients only when the disease is onset at early-stage and at the level of non-metastasis. Advanced-stage breast cancer with metastasis in distant organs is assumed to be non-curable with the currently available therapeutic options (2). As far as the survival of advanced breast cancer patients is concerned, which can be manageable by therapy, but the primary goal is only to prolong survival by controlling symptoms by introducing minimum-treatment associated toxicity (2). Based on the molecular expression of cell membrane receptors, the onset of breast cancer can be classified into four subtypes such as normal-like expression of receptors, molecular expression of cell surface estrogen receptor-positive (luminal), human epidermal growth factor receptor 2 (HER2) enriched, and basal-like cell surface receptors (3). Another type of cancer discovered among breast cancer patients is the triple-negative breast cancer (TNBC), which generally occurs in about 10-15% of patients with breast cancers (4). The term triple-negative breast cancer is denotes the absence of molecular target expressions such as estrogen, progesterone, and a1HER2. The onset TNBC was commonly reported in women younger than age 40 years and women reported to have a *BRCA1* mutation (5). Interestingly, the onset of TNBC was reported to grow faster than other breast cancer types, and the therapeutic options for managing patients with TNBC are limited and largely unexplored (4, 5). The suppressor of cytokine signaling 1 (SOCS1), a prototype molecule of the SOCS family, was recognized in the beginning as a negative feedback regulator of cytokine signaling. Still, in recent years, its mode of action and associated mechanisms have been studied. Recent studies point out that SOCS1 performs different functions in different cell types. In tumor cells, its mode of action and function during carcinogenesis seems to be complicated, complex, and highly controversial (6). Several studies reported abnormal expression of SOCS1 in several cancer cells, whereas studies also reported inhibition of SOCS1 expression at the mRNA level due to hypermethylation in other cancer cell types (6). In order to inhibit or restore SOCS1 expression in different cancer cell types, several opposite strategies were applied. Some studies point out that silencing of SOCS1 in cancer cells improves the sensitivity towards IFNs and suppresses cancer cell proliferation. In contrast, other studies reveal that the drugs perform demethylation to restore SOCS1 expression of SOCS1 in cancer cells (6, 7). These contradictory findings in different cancer cell types may be due to the onset of different tumors at different origins, and these studies clearly indicate that SOCS1 warrants its role in cancer onset and it might be a novel diagnostic or prognostic biomarker for cancer therapies (6, 8). MicroRNAs (miRNAs) are one of the most abundant classes of small RNA nucleotides known to regulate the genes by complementary pairing with their 3'untranslated (3'UTR) at the post-transcriptional level. Now it has been established that miRNAs regulate 70-80% of all human protein-coding genes and these small nucleotides RNA sequences have been involved in almost all cellular events including proliferation, migration and invasion of normal and pathogenic tissues (9, 10). The role of miRNAs as key regulators of the number of pathogenic genes has been well-known in numerous human disorders, including breast cancer (11–13). Numerous studies have provided evidence that miR-155 exhibits substantial up-regulation in breast cancer and has a close association with clinic-pathological markers, tumor stage, sensitivity to radiotherapy and chemotherapy, as well as poor survival rates (14). Additionally, research indicates that miR-155 significantly boosts the growth and movement of breast cancer cells, potentially acting as an oncogene in the disease (14–16). Therefore, there is a preliminary belief that miR-155 plays a vital role in the proliferation and migration of breast cancer cells by reducing the expression of SOCS1 through its targeting of the 3`UTR of SOCS1 mRNA sequence (14–16). Nanotechnology has become an attractive area in medical science. The application of engineered nanoparticles as a therapeutic tool in multiple human diseases, such as cancer, has been widely explored and offered in treatment and prevention (17). In the recent past, engineered nanoparticles have shown a tremendous therapeutic perspective as anticancer agents with numerous treatment approaches. They can be used as molecular probes, anti-angiogenic, antitumor, anti-permeability, and antiproliferative (18–20). Among all studied chemically synthesized nanoparticles, gold nanoparticles (AuNPs) have attracted the most therapeutic attention for several potential reasons, such as easy chemical synthesis, excellent biocompatibility, and convenient coupling with biologically active compounds (19, 21). Recently, the use of AuNPs as nanomedicine for cancer treatment has gathered global attention, but the therapeutic mechanism(s) behind their mode of action has largely been unexplored (22–24). In this study, we hypothesized that engineered AuNPs coupled with citrate inhibit the IFN-γ-induced carcinogenesis by modulating the expression of miRNAs in triple-negative breast cancer patients. To test this hypothesis, AuNPs conjugated with citrate (citrate-AuNPs) were newly synthesized using trisodium citrate method and characterized by UV-Vis spectroscopy and dynamic light scattering studies. The therapeutic potential of these citrate-AuNPs was evaluated on IFN-γ-induced SOCS1 expression on triple-negative breast cancer cells MDA-MB-231. The Targetscan bioinformatics approach was used to screen 3'UTR of SOCS1 mRNA to determine seed matched sequences complementary to miRNAs sequences. The role of specific miRNA in the abnormal regulation of pathogenic genes was studied by transfection of cancer cells with specific miRNA inhibitors. Our novel data showed citrate-AuNPs inhibit the SOCS1 expression via modulating the expression of microRNA-155-5p in MDA-MB-231 cells stimulated IFN-γ. These are novel findings that have never been reported before and may be of value in the design of novel therapies for treating triple negative breast cancer patients.

## Methods

### Synthesis of citrate gold nanoparticles

The citrate gold nanoparticles (citrate-AuNPs) were prepared by the chemical method described previously (25, 26) with some modifications. The 26.2 mM trisodium citrate (Na_3_C_6_H_5_O_7_^.2^H_2_O) solution was prepared in milli-Q water. At the same time, 25 ml of milli-Q water was boiled at 100°C, and 10 ml of 2 mM chloroauric acid (HAuCl_4_) was added. After boiling of chloroauric acid, 4 ml of 26.2 mM trisodium citrate solution was added, and all together, these solutions were boiled for 1.5 hours and then allowed to cool at room temperature. After cooling, the whole solution was centrifuged for a few minutes, and the supernatant was stored at 4°C and used as citrate gold nanoparticles.

### Ultraviolet-visible spectrophotometry and dynamic light scattering studies

Ultraviolet-Visible (UV-Vis) spectrophotometry was used to characterize the newly synthesized citrate-AuNPs using Lambda XLS Perkin Elmer UV-Vis spectrophotometer (PerkinElmer, Inc., MA, USA) as described previously (27). The particle size, PDI, and zeta-potential of chemically synthesized citrate-AuNPs were measured by Dynamic Light Scattering (DLS) using Malvern Zetasizernano 6.01 (Herrenberg, Germany) as previously described (24, 25). The timing of sampling was automatically set on the instrument. For every 10 sub-runs, three measurements were allowed, and all calculations were performed in accordance with the previously published studies (24, 25).

### MDA-MB-231 cancer cells culture

A triple-negative human breast adenocarcinoma derived MDA-MB-231 cell line was grown and maintained in high glucose DMEM medium (catalog # SLM-120-B, Millipore) supplemented with 10% heat-inactivated fetal bovine serum (FBS) penicillin (100 U/ml), streptomycin (100 μg/ml) at 37°C in 5% CO_2_ and 95% air as described previously (27, 28).

### Treatment of MDA-MB-231 cells with citrate-NPs or interferon-γ

Overnight serum-starved MDA-MB-231 cells (70–80% confluent) were treated with citrate-AuNPs in different experimental conditions, and the viability of MDA-MB-231 was examined by Cell Titer-Glo Luminescent Cell Viability Assay kit (catalog # G7573, Promega, Wl, USA) as described previously (29). In other sets of experiments, serum-starved MDA-MB-231 cells were pretreated with citrate-AuNPs (0.2-0.4 µg/ml) for 2 hours and then stimulated with 0.1 µg/ml of interferon-λ (IFN-γ, EMD Millipore Corporation, Temecula, CA, USA). In contrast, as described previously, the MDA-MB-231 cells cultured without citrate-AuNPs or IFN-λ served as a negative control (30).

### Bioinformatics approach for the prediction of microRNAs complementary to 3’UTR of SOCS1 mRNA

To predict microRNAs complementary to 3'UTR of SOCS1 mRNA, TargetScan bioinformatics algorithm (http://www.targetscan.org/) was used as described previously (31).

### Transfection of MDA-MB-231 cells with miRNA inhibitors

Human cancer cells were transfected with anti-miRNAs or miRNA inhibitors (100 nM; Ambion/Qiagen) using HiPerfect Transfection Reagent (Qiagen, USA) as described previously (32). After 72 h post-transfection, MDA-MB-231 cells were pretreated with citrate-AuNPs (0.2-0.4 µg/ml) for 2 h or stimulated with IFN-λ (0.1µg/ml) for 0.5-6 h to determine the expression of miRNA, mRNA or protein.

### Luciferase reporter assays

Luciferase reporter assays were performed to validate binding of miR-155-5p in SOCS1 3'-untranslated region (`UTR) using luciferase assay kit (Promega, WI, USA). A luciferase reporter vector having the entire 3′UTR of SOCS1 mRNA (# ENST00000332029.2) and an empty vector containing only luciferase gene and active promoter (Applied Biological Materials Inc., BC, Canada or Switch Gear Genomics, CA, USA) were used in the reporter assays. MDA-MB-231 cells were co-transfected with a reporter plasmid (100 nM), miRNA inhibitor (50-100 nM), or negative control miRNAs using HiPerfect Transfection Reagent (Qiagen). After 24 h post-transfection, MDA-MB-231 cells were treated with citrate-AuNPs (0.2-0.4 µg/ml), and dual luciferase activity was measured as described previously (32, 33).

### MicroRNA preparation, complementary DNA synthesis, and quantitative real-time PCR

Ambion mirVana miRNA isolation kit (Ambion, Foster City, CA, USA) was used to isolate total RNA (0.6-1.0 µg), including miRNA fraction from experimental MDA-MB-231 cancer cells in accordance with the manufacturers' instructions. The first strand cDNA samples were synthesized from total RNA by Superscript First Strand cDNA synthesis kit (Applied Biosystems). The specific TaqMan assays (Applied Biosystems) were used to quantify the expression of mRNAs and miRNAs using Applied Biosystem real-time PCR instruments (StepOne real-time PCR system, Life Technologies, Foster City, CA, USA). Expression of GAPDH or RNU6B was used as an endogenous control for the quantification of mRNA or miRNA, respectively. The SOCS1 (NM_003745) expression primers were forward sequence 5'TTCGCCCTTGCGTGAAGATGG3` and Reverse sequence 5`TAGTGCTCCAGCAGCTCGAAGA3`. Whereas for GAPDH (NM_001256799), the forward sequence was 5'GTCTCCTCTGACTTCAACAGCG3`, and the reverse sequence was 5`ACCACCCTGTTGCTGTAGCCAA3`. As described previously, the double delta CT method was used to quantify the relative expression of desired mRNA and miRNA (34, 35).

### Western immunoblotting and densitometric analysis

Western immunoblotting was used to quantify protein levels in experimental MDA-MB-231 cells, as described previously (36). Briefly, the cell lysates of transfected or non-transfected MDA-MB-231 cells were prepared using the Pierce RIPA cell lysis buffer (catalog # 89901, Thermo Scientific, IL, USA). They were loaded on 10% resolving SDS-PAGE with 2.5% stacking and western blots were analyzed with probing of specific primary antibodies (Cell Signaling Technology Beverley, MA, USA). Images were captured using Syngene G-BOX Chemi XRQ system (Beacon House, Cambridge, UK). Moreover, images were further analyzed densitometrically using the UNSCAN-IT (Silk Scientific Corporation, Utah, USA). Each band was scanned 3 times with normalization of correction background and the data were presented in average pixel band ratios as described previously (37).

### Transcription factor NF-kappa B p65 and NF-kappa B p50 assays

Activated transcription factors NF-κBp65 and NF-κBp50 in nuclear extracts of transfected or non-transfected MDA-MB-231 cells were determined using a highly sensitive Transcription Factor Assay Kit (catalog # ab133128, Abcam, England). Transcription Factor NF-κBp65 combo positive control (Abcam) and Transcription Factor NF-κBp50 combo positive control (Abcam) were also used as experimental controls. The plates with the final reaction were read at 450 nm using an automatic Zenythmicroplate reader (Anthos 3100, Salzburg, Austria). The nuclear extract of transfected or non-transfected MDA-MB-231 cells was prepared as described previously (37, 38).

### Statistical analysis

Statistical comparisons among the tested groups were performed by One-way ANOVA analysis followed by Tukey’s *post-hoc* analysis or Two-way ANOVA followed by Bonferroni *post-hoc* tests using Graph Pad Prism-5 (San Diego, CA, USA). p<0.05 was considered significant.

## Results

### Characterization and stability of chemically engineered citrate gold nanoparticles

**Figure 1:**
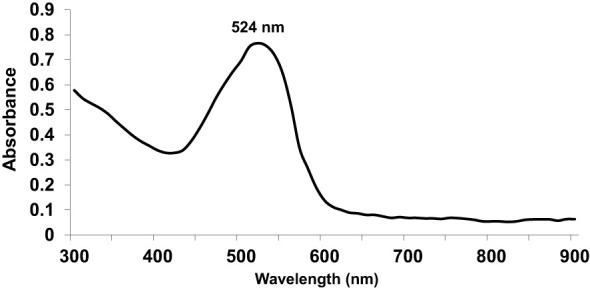
Ultraviolet-Visible (UV-Vis) absorption spectra of gold nanoparticles coupled with citrate (citrate-AuNPs).The peak of surface plasmon resonance of chemically synthesized citrate-AuNPs was found at λ_max_ 524 nm. The citrate-AuNPs samples were in milli-Q ultra-pure water and UV-Vis spectra was recorded at room temperature. A single spectrum represents a mean of five independent experiments of chemically synthesized citrate-AuNPs.

**Figure 2:**
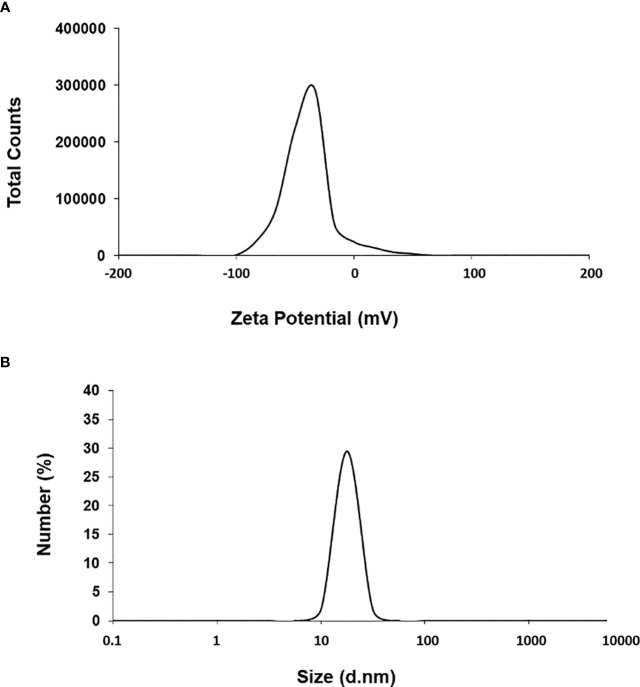
Zeta-potential **(A)** and particle size distribution **(B)** of citrate-AuNPs determined by dynamic light scattering. Aqueous samples of citrate-AuNPs samples were used in recording of measurements of dynamic light scattering. Each spectrum represents a mean of five independent experiments of chemically synthesized citrate-AuNPs.

**Figure 3:**
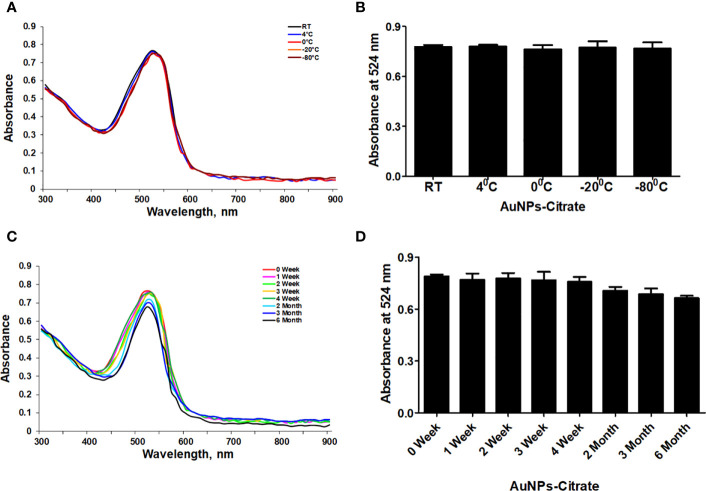
Stability determination of chemically synthesized citrate-AuNPs. **(A)** The citrate-AuNPs samples were stored at indicated temperatures and the storage stability was determined by UV-Vis spectroscopy. **(B)** Storage stability of nanoparticles samples against indicative temperatures was determined by an observed λmax of citrate-AuNPs. **(C)** The citrate-AuNPs samples were stored at 4°C for 0 week to 6 month and the stability against storage time was determined by UV-Vis spectroscopy. **(D)** Stability of nanoparticles samples against storage time periods was determined by an observed λmax of citrate-AuNPs. Aqueous solution of citrate-AuNPs samples were stored at 4°C. Each observation represents a mean of five independent experiments of chemically synthesized citrate-AuNPs.

First, we used UV-Vis spectrophotometry for the analysis of chemically synthesized citrate-AuNPs. The UV-Vis spectra of newly synthesized citrate-AuNPs showed a single peak with maximum wavelength (λmax) at 524 nm. **Figure 1** shows the peak of surface plasmon resonance of chemically synthesized citrate-AuNPs at 524 nm. The stability of chemically synthesized citrate-AuNPs was determined by quantifying zeta potential, particle size, and a polydispersity index (PDI). The zeta potential of citrate-AuNPs was found to be -32.2 mV indicating the stability of citrate-AuNPs in nature and distribution of negative charge in their surface (**Figure 2A**). The size distribution of citrate-AuNPs quantified using DLS and the average size was found to be 28.3 nm (**Figure 2B**) and PDI value was obtained at 0.435, indicating the low polydispersity nature of chemically synthesized citrate-AuNPs. Moreover, the storage stability of citrate-AuNPs was also investigated by UV-Vis spectroscopy. The citrate-AuNPs in different aliquots were stored for 2 weeks at room temperature (RT), 4°C, 0°C, -20°C and -80°C varying temperatures and UV-Vis spectra were taken. All aliquots of citrate-AuNPs samples showed similar UV-vis spectra with maximum absorbance at 524 nm (**Figure 3A**). These results were further verified by taking absorbance at 524 nm of all citrate-AuNPs aliquots and the average absorbance of all citrate-AuNPs samples remains the same (**Figure 3B**). To investigate the storage stability of citrate-AuNPs in more detail, samples of citrate-AuNPs were stored at 4°C for 0 weeks to 6 months and the UV-Vis spectra were taken (**Figure 3C**). The peak of UV-Vis spectra of citrate-AuNPs samples from 0 weeks to 3 weeks almost the same, then the peak absorbance started declined from 4 weeks to 6 months but this decline in the absorbance was insignificant (p>0.05). These results were further re-validated by taking the absorbance at 524 nm, and the obtained data again showed the decrease in the absorbance from 4 weeks to 6 months (p>0.05; **Figure 3D**). These results point out that the chemically synthesized citrate-AuNPs was highly stable up to 3 weeks at 4°C.

### Cytotoxicity check of citrate-AuNPs on the viability of MDA-MB-231 cells *in-vitro*

**Figure 4:**
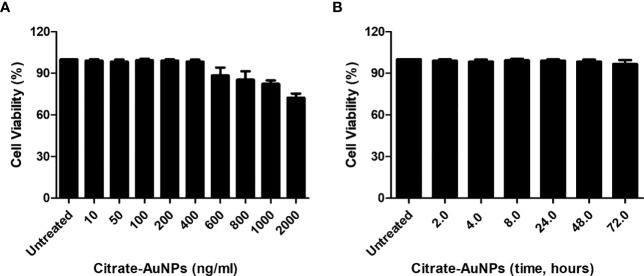
Effect of citrate-AuNPs on the viability of an epithelial, human breast cancer cell line MDA-MB-231 cells. **(A)** Viability of MDA-MB-231 cells (3×10^6^ cells/ml) against indicated concentration of chemically synthesized citrate-AuNPs. The MDA-MB-231 cancer cells were treated with 10 ng/ml to 2000 ng/ml of citrate-AuNPs for 24 h. **(B)** Viability of MDA-MB-231 cells (3×10^6^ cells/ml) against indicative treatment time of citrate-AuNPs. The MDA-MB-231 cancer cells were treated with 0.4 µg/ml of citrate-AuNPs for 2 - 72 h. The citrate-AuNPs treated MDA-MB-231 cancer cells were incubated in serum starved DMEM culture medium at 37°C in 5%CO_2_ and the viability was determined by the Cell Titer-Glo Luminescent Cell Viability Assay kit (Promega Corporation, WI, USA).

The cytotoxicity of chemically synthesized citrate-AuNPs was checked by measuring the viability of MDA-MB-231 cells upon the treatment. The serum-starved cancer cells MDA-MB-231 were treated with 10-2000 ng/ml of citrate-AuNPs for 24 h. The viability of these treated cells was estimated by Cell Titer-Glo Luminescent Cell Viability Assay kit (Promega Corporation, WI, USA). As shown in **Figure 4A**, the percent cell viability was very stable up to 400 ng/ml (0.4 µg/ml) of citrate-AuNPs as compared with untreated cells (P>0.05) and then started declining on the further increase of citrate-AuNPs concentration. Effect of treatment time of citrate-AuNPs on the viability of MDA-MB-231 cells was also tested by incubation of citrate-AuNPs (0.4 µg/ml) for 2-72 h in serum starved MDA-MB-231 cells and viability of these cells was determined. The chemically synthesized citrate-AuNPs (0.4 µg/ml) were found to be non-toxic for MDA-MB-231 cells (**Figure 4B**). Based on these results, the maximum concentration of citrate-AuNPs used in further studies was 0.4 µg/ml.

### TargetScan bioinformatics algorithm for the duplex formation of hsa-miR-155-5p in 3'UTR of human SOCS1 mRNA (NM_003745)

**Figure 5:**
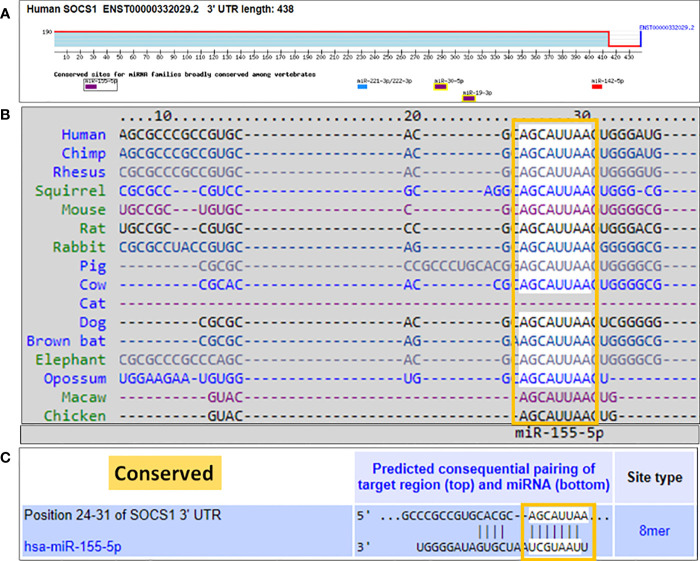
Bioinformatics analysis of seed sequence of hsa-miR-155-5p in 3’UTR of human SOCS1 mRNA. **(A)** 3`UTR of human SOCS1 mRNA (ENST00000332029.2) shows conserved site of hsa-miR-155-5p. **(B)** TargetScan prediction of conversed sequence of miR-155-5p in 3’UTR of SOCS1 mRNA in cross-species including human. **(C)** Predicted duplex of hsa-miR-155-5p with the seed matched sequence in the 3’UTR of human SOCS1 mRNA. The sequences show in orange rectangle are the potential locations for the formation of miRNA-mRNA duplex.

We used a computer-based algorithm to determine the possible binding of microRNA hsa-miR155-5p with the complementary sequence in the 3'UTR of human SOCS1 mRNA. TargetScan algorithm determined that human 3'UTR of SOCS1 mRNA (ENST00000332029.2) contains 438 nucleotide bases and has a conserved site for hsa-miR155-5p in between 24-31 nucleotide bases (**Figure 5A**). The conserved sequence of miR155-5p in the 3'UTR of SOCS1 mRNA is not only predicted in human, but also other species such as chimp, rhesus, squirrel, mouse, rat, rabbit, pig, cow, cat, dog, brown bat, elephant, opossum, macaw and chicken (**Figure 5B**). The exact location of complementary pairing between 3'UTR of human SOCS1 mRNA and hsa-miR155-5p has been shown in **Figure 5C**.

### Validation of consequential pairing of hsa-miR155-5p with 3'UTR-SOCS1 mRNA

**Figure 6:**
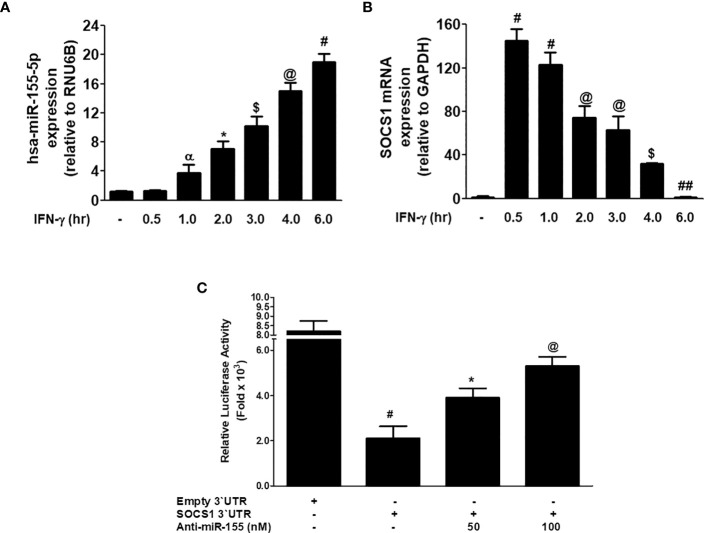
Inverse co-relation of microRNA-155-5p with SOCS1 expression. **(A)** Expression of hsa-miR155-5p in MDA-MB-231 cancer cells (3×10^6^ cells/ml) stimulated with IFN-γ (0.1 µg/ml) for indicative time determined by TaqMan assays. Unstimulated MDA-MB-231 cells were used as controls and expression of RNU6Bwas used as an endogenous control. The data shown are mean ± SD of three independent experiments. The data differ without a common symbol, p<0.05. **(B)** SOCS1 mRNA expression determined by TaqMan assay. Unstimulated MDA-MB-231 cells were used as controls and expression of GAPDH was used as an endogenous control. The data shown are mean ± SD of three independent experiments. The data differ without a common symbol, p<0.05. **(C)** Luciferase activity in MDA-MB-231 cells transfected with the reporter vector containing entire 3’UTR of SOCS1 mRNA (SOCS1 3’UTR) and anti-miR155-5p. Transfection of MDA-MB-231 cells with Empty 3’UTR vector (vector containing only luciferase gene and active promoter) alone and SOCS1 3’UTR alone was used as a negative and positive control, respectively. The data shown are mean ± SD of three independent experiments. The data differ without a common symbol, p<0.05.

The correlation between miR-155-5p and SOCS1 mRNA was investigated in MDA-MB-231 cancer cells stimulated with IFN-γ. Stimulation of MDA-MB-231 cells with 0.1µg/ml IFN-γ for 0.5 h to 6.0 h caused the sequential increase of hsa-miR155-5p expression, and the maximum increase was observed at 6 h (p<0.05; **Figure 6A**). Whereas, treatment of MDA-MB-231 cells with 0.1µg/ml IFN-γ for 0.5 h caused a sharp increase in SOCS1 at both mRNA (p<0.0001) and protein (P<0.001) levels. And then, there was a sequential decline in the expression of SOCS1 at both mRNA from 0.5 - 6 h treatment of IFN-γ (**Figure 6B**). These results show that treatment of MDA-MB-231 cells with IFN-γ (0.1 µg/ml) for 0.5 h showed minimum expression of hsa-miR155-5p and maximum SOCS1 mRNA. This inverse correlation between hsa-miR155-5p and SOCS1 was validated by a reported luciferase assay using co-transfection of MDA-MB-231 cells with a reporter clone containing entire 3'UTR of SOCS1 (SOCS1 3'UTR) and miR-155-5p inhibitor (anti-miR155). A significant increase in luciferase activity was observed in MDA-MB-231 cells co-transfected with SOCS1 3'UTR and anti-miR155 compared with those MDA-MB-231 cells transfected with SOCS1 3'UTR alone (p<0.05). The transfection of MDA-MB-231 cells with SOCS1 3'UTR alone significantly reduced the relative luciferase activity as compared with those MDA-MB-231 cells transfected with empty `UTR (p<0.0001). The transfection of MDA-MB-231 cells with reporter clones of SOCS1 3'UTR alone and empty 3'UTR alone was used as positive and negative controls, respectively. These results confirmed the validation of the pairing of hsa-miR-155-5p with the seed-matched sequence present in the 3'UTR of human SOCS1 mRNA (**Figure 6C**).

### Citrate-AuNPs inhibit expression of hsa-miR-155-5p, SOCS1 mRNA and protein expression in IFN-γ-stimulated MDA-MB-231 cancer cells

**Figure 7:**
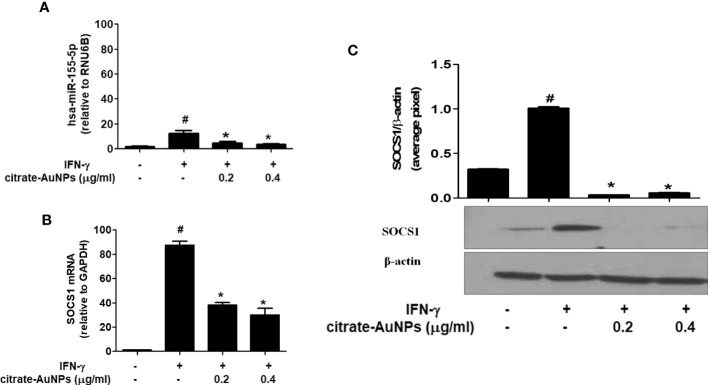
Effect of citrate-AuNPs on the SOCS1 expression in IFN-γ-stimulated MDA-MB-231 cancer cells. **(A)** Relative hsa-miR-155-5p expression determined by double delta CT method using quantitative PCR. The expression of RUN6B was used as an endogenous control. MDA-MB-231 cells (3×10^6^ cells/ml) were pretreated with citrate-AuNPs (0.2-0.4μg/ml) for 2 h and stimulated by IFN-γ for 60 minutes. The data shown are mean ± SD of three independent experiments. The data differ without a common symbol, p<0.05. **(B)** Relative SOCS1 mRNA expression determined by double delta CT method using quantitative PCR. The expression of GAPDH was used as an endogenous control. MDA-MB-231 cells (3×10^6^ cells/ml) were pretreated with citrate-AuNPs (0.2-0.4μg/ml) for 2 h and stimulated by IFN-γ for 60 minutes. The data shown are mean ± SD of three independent experiments. The data differ without a common symbol, p<0.05. **(C)** SOCS1 protein expression determined by western blotting. The expression of β-actin was used as an endogenous control. MDA-MB-231 cells (3×10^6^ cells/ml) were pretreated with citrate-AuNPs (0.2-0.4μg/ml) for 2 h and stimulated by IFN-γ for 60 minutes. Images of bands were captured digitally using the Un-Scan-It software and were presented as average pixels after normalization with β-actin band intensities. The data shown are mean ± SD of three independent experiments. The data differ without a common symbol, p<0.05.

The potential of chemically synthesized citrate-AuNPs was evaluated on the MDA-MB-231 cancer cells stimulated IFN-γ. Treatment of MDA-MB-231 cells with citrate-AuNPs (0.2-0.4 µg/ml) prior to the stimulation with IFN-γ, significantly inhibited expression of hsa-miR-155-5p (**Figure 7A**) and the SOCS1 mRNA (**Figure 7B**) in a dose-dependent manner as determined by quantitative RT-PCR (p<0.05). To further verify the potential of citrate-AuNPs on the SOCS1 protein expression, the cell lysates from MDA-MB-231 cells treated with citrate-AuNPs and IFN-γ were analyzed by western immunoblotting using the primary antibodies specific for SOCS1. As shown in the **Figure 7C**, pre-treatment with 0.2-0.4 μg/ml of citrate-AuNPs significantly reduced the IFN-γ-induced SOCS1 protein expression in a dose-dependent manner (p<0.05) in activated MDA-MB-231 cells.

### Citrate-AuNPs inhibits SOCS1 expression through hsa-miR155-5p and 3`UTR of SOCS1 mRNA in MDA-MB-231 cells

**Figure 8:**
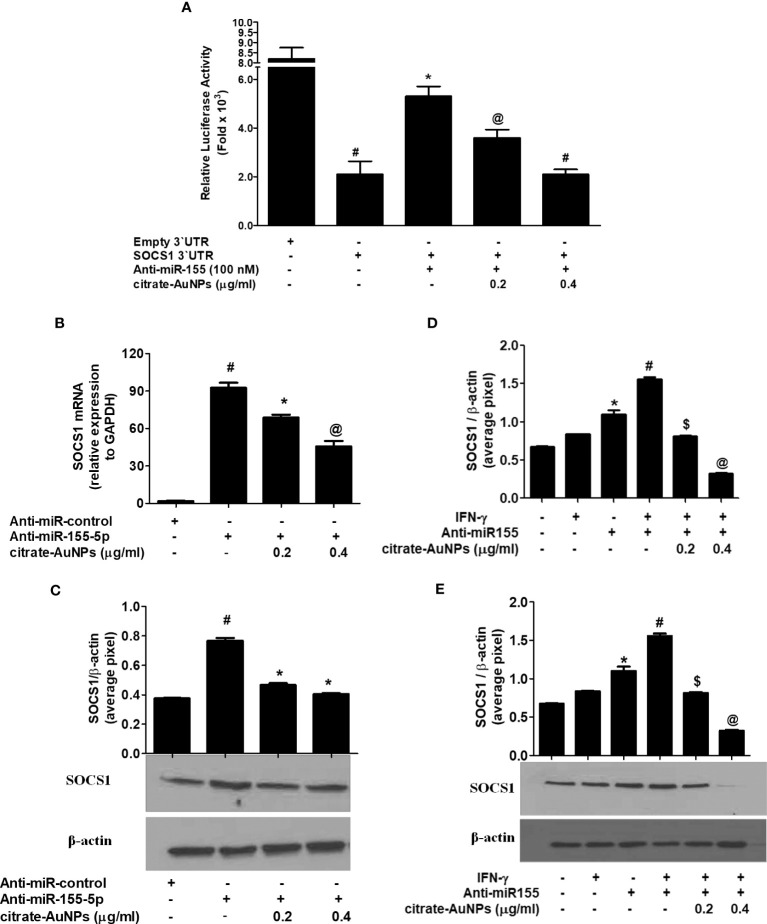
Citrate-AuNPs inhibits SOCS1 expression via modulation of hsa-miR155-5p in MDA-MB-231 cells. **(A)** Effect of citrate-AuNPs on the luciferase activity in MDA-MB-231 cells co-transfected with the SOCS1 3’UTR reporter vector and anti-miR155-5p. Transfection of MDA-MB-231 cells with empty 3’UTR vector alone and SOCS1 3’UTR alone was used as negative and positive controls, respectively. ^#^p<0.0001 vsMDA-MB-231 cells transfected with Empty 3’UTR vector; ^@^p<0.05vs ##; ^#^p<0.01 vs ##. **(B)** Effect of citrate-AuNPson SOCS1 mRNA expression in MDA-MB-231 cells transfected with anti-miR155-5p. Expression of GAPDH was used as endogenous control. ^#^p<0.001 vsMDA-MB-231 cells transfected with anti-miR-control; ^*^p<0.05 vs #; ^@^p<0.001 vs #. **(C)** Effect of citrate-AuNPson SOCS1 protein expression in MDA-MB-231 cells transfected with anti-miR155-5p determined by western blotting. Expression of β-actin was used as endogenous control. MDA-MB-231 cells transfected with anti-miR-control; ^*^p<0.05 vs #. Images of bands were captured digitally using the Un-Scan-It software and were presented as average pixels after normalization with β-actin band intensities. The data shown are mean ± SD of three independent experiments. **(D)** Effect of citrate-AuNPs on IFN-γ-induced SOCS1 mRNA expression in MDA-MB-231 cells transfected with anti-miR155-5p. Expression of GAPDH was used as endogenous control. *p<0.001 vs untreated MDA-MB-231 cells; ^*^p<0.05 vs #; ^@^p<0.001 vs #. **(E)** Effect of citrate-AuNPs on IFN-γ-induced SOCS1 protein expression in MDA-MB-231 cells transfected with anti-miR155-5p. Expression of β-actin was used as endogenous control. *p<0.001 vs untreated MDA-MB-231 cells; ^*^p<0.05 vs #; ^#^p<0.001 vs $; ^#^p<0.001 vs @. Images of bands were captured digitally using the Un-Scan-It software and were presented as average pixels after normalization with β-actin band intensities. The data shown are mean ± SD of three independent experiments.

Inhibition of SOCS1 expression by citrate-AuNPs via involvement of 3'UTR of SOCS1 and hsa-miR155-5p was validated by luciferase reporter assays. The results in **Figure 8A** showed a marked increase in the relative luciferase activity in MDA-MB-231 cells, co-transfected with SOCS1 3'UTR reporter and anti-miR155-5p (p<0.01). This increase of luciferase activity was completely reversed by the treatment of these co-transfected cancer cells with citrate-AuNPs (**Figure 8A**). This reduction in the luciferase activity by citrate-AuNPs indicates that these nanoparticles negatively co-regulate the expression of SOCS1 and hsa-miR-155-5p. Therapeutic potential of citrate-AuNPs against SOCS1 expression via hsa-miR155-5p was further tested by transfection with anti-miR155 and then treated with citrate-AuNPs. As shown in **Figure 8B**, transfection of MDA-MB-231 cells with anti-miR155-5p significantly increases the expression of SOCS1 mRNA as compared to those sets of cells transfected with anti-miR-control (p<0.001). Interestingly, this marked increase of SOCS1 mRNA was significantly inhibited by citrate-AuNPs in a dose-dependent manner (p<0.05). Furthermore, we also determine whether inhibition of SOCS1 expression at mRNA level also affects the protein level. As expected, treatment of anti-miR155-transfected cancer cells with citrate-AuNPs also inhibited SOCS1 expression at the protein level (p<0.05; **Figure 8C**). These results confirmed that citrate-AuNPs inhibited SOCS1 expression via modulation of hsa-miR155-5p expression. Therapeutic effects of citrate-AuNPs on the normalization of upregulated expression of SOCS1 via hsa-miR155-5p was further re-validated by transfection of MDA-MB-231 cells with anti-miR155-3p followed by co-treatment with citrate-AuNPs and IFN-γ. As shown in **Figure 8D**, stimulation of anti-miR155-transfected MDA-MB-231 cells with IFN-γ, significantly enhanced the SOCS1 mRNA expression compared to non-transfected cancer stimulated with IFN-γ (p<0.05). Interestingly, treatment with citrate-AuNPs significantly inhibited IFN-γ-induced SOCS1 mRNA in a dose-dependent manner in MDA-MB-231 cells transfected with anti-miR155-5p (p<0.05). To determine whether these modulations of SOCS1 mRNA expression also affected at the protein level, cell lysates of these transfected or non-transfected MDA-MB-231 cells were analyzed by western blotting using specific antibodies for SOCS1. Western blot analysis showed that treatment with citrate-AuNPs significantly inhibited IFN-γ-induced SOCS1 protein expression in a dose-dependent manner in MDA-MB-231 cells transfected with anti-miR155-5p (**Figure 8E**; p<0.05). The data clearly indicated that chemically synthesized citrate-AuNPs reduce SOCS1 mRNA/protein expression via modulation of hsa-miR155-5p expression.

### Citrate-AuNPs inhibits IFN-γ-induced NF-κB activation via hsa-miR155-5p in MDA-MB-231 cells

**Figure 9:**
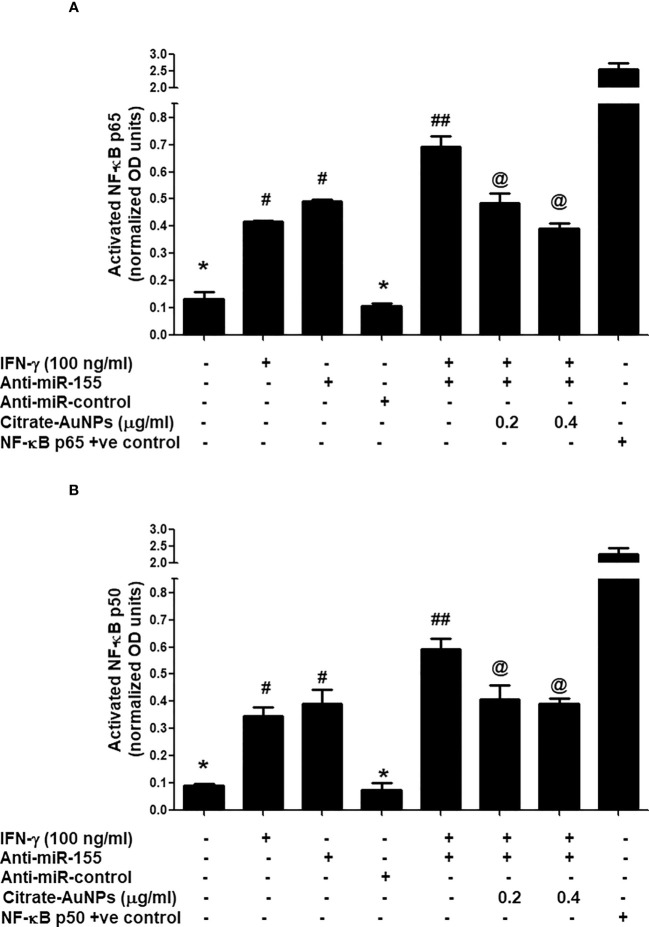
Citrate-AuNPs inhibits IFN-γ induced transcription factor NF-κB activation via hsa-miR155-5p in MDA-MB-231 cells transfected or non-transfected with anti-miR155. **(A)** Citrate-AuNPs inhibits IFN-γ-induced NF-κB p65 activation in MDA-MB-231 cells transfected or non-transfected with anti-miR155determined by NF-κB p65 transcription factor kit (Abcam). The NF-κB p65 positive control was supplied with the kit. The data shown are mean ± SD of three independent experiments. The data differ without a common symbol, p<0.05. **(B)** Citrate-AuNPs inhibits IFN-γ-induced NF-κB p50 activation in MDA-MB-231 cells transfected or non-transfected with anti-miR155determined by NF-κB p50 transcription factor kit (Abcam). The NF-κB p50 positive control was supplied with the kit. The data shown are mean ± SD of three independent experiments. The data differ without a common symbol, p<0.05.

Inhibition of IFN-γ-induced NF-κB activation by citrate-AuNPs via involvement of 3'UTR of SOCS1 and hsa-miR155-5p was studied by transfection of MDA-MB-231 cells with anti-miR155-5p. The results in **Figure 9A** showed a marked increase of NF-κB p65 levels in the nuclear extract of MDA-MB-231 cells treated with IFN-γ alone for 30 minutes as compared with untreated MDA-MB-231 cells (p<0.01). Similarly, a marked increase of NF-κB p65 levels in the nuclear extract was also observed in MDA-MB-231 cells transfected with anti-miR155-5p compared to those MDA-MB-231 cells transfected with anti-miR-control (p<0.01). Interestingly, a sharp enhancement in activated NF-κB p65 was noticed when transfected MDA-MB-231 cells with anti-mR155-5p were stimulated with IFN-γ (p<0.001). Significantly, this increase of NF-κB p65 was reversed by citrate-AuNPs treatment in a dose-dependent manner. This reduction in the NF-κB p65 activation by citrate-AuNPs indicates that these nanoparticles inhibit NF-κB p65 activity *via* modulation of hsa-miR-155-5p expression. Therapeutic potential of citrate-AuNPs against NF-κB p50 activation via hsa-miR155-5p was further tested by transfection with anti-miR155 and then treated with citrate-AuNPs. The results in **Figure 9B** showed a marked increase of NF-κB p50 levels in the nuclear extract of MDA-MB-231 cells treated with IFN-γ alone for 30 minutes compared with untreated MDA-MB-231 cells (p<0.01). Similarly, a marked increase of NF-κB p50 levels in the nuclear extract was also observed in MDA-MB-231 cells transfected with anti-miR155-5p compared to those MDA-MB-231 cells transfected with anti-miR-control (p<0.01). Interestingly, a sharp enhancement in activated NF-κB p50 was noticed when transfected MDA-MB-231 cells with anti-mR155-5p were stimulated with IFN-γ (p<0.001). Importantly, this increase of NF-κB p50 was reversed by citrate-AuNPs treatment in a dose-dependent manner. This reduction in the NF-κB p50 activation by citrate-AuNPs indicates that these nanoparticles inhibit NF-κB p50 activity via modulation of hsa-miR-155-5p expression. Taken together, the data showed that the chemically synthesized citrate-AuNPs inhibit SOCS1 expression via modulation of hsa-miR155-5p and deactivation of NF-κB p65/p50 activity.

## Discussion

**Figure 10:**
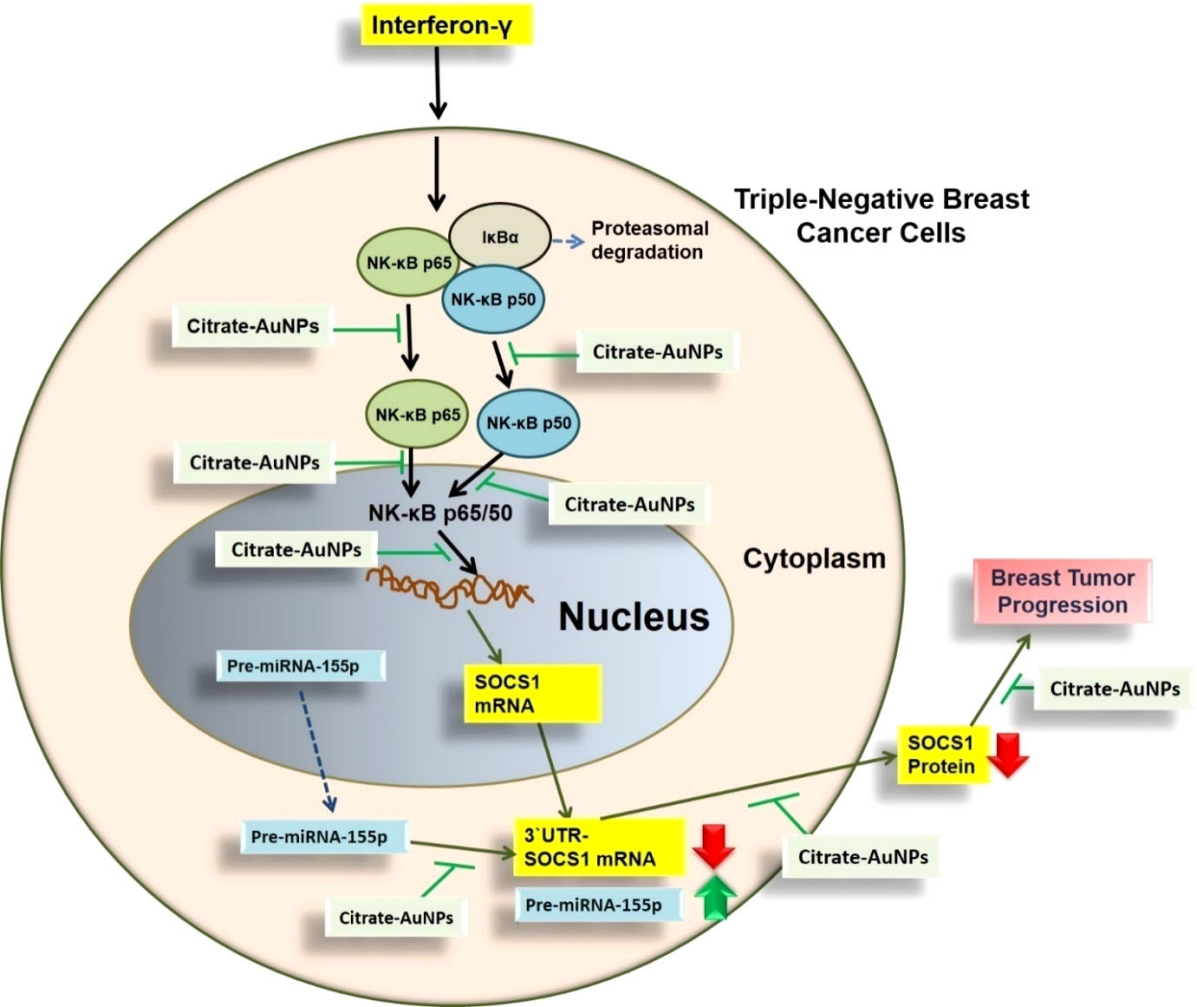
Schematic representation illustrating the therapeutic potential of AuNPs investigated in this study. IFN-γ activated NF-κB mediated signaling in TNBC cells through SOCS1is inhibited by AuNP via negative regulation of has-miR155-5p. All possible targets of AuNPs induced inhibition to SOCS1 are systematically demonstrated. NF-κB complex was demonstrated by NF-κB p65 subunit and the pathways shown with broken arrows were not investigated. AuNPs, gold nanoparticles; SOCS1,suppressor of cytokine signaling 1; NF-κB, nuclear transcription factor-kappa B; 3’UTR-SOCS1 mRNA 3’ untranslated region - suppressor of cytokine signaling 1 mRNA.

This is the first report that shows that the chemically engineered gold nanoparticles conjugated with citrate inhibit IFN-γ induced SOCS1 mRNA/protein expression via modulating miRNA-155-5p in triple-negative human breast cancer cells (**Figure 10**). Interferons (IFNs) play a significant role in breast cancer pathogenesis, influencing both tumor progression and the immune response against cancer cells (39, 40). IFNs are cytokines released by immune cells in response to viral infections and other cellular stressors. In breast cancer, IFNs can exert both anti-tumor and pro-tumor effects depending on the context (41). On one hand, IFNs activate immune cells such as natural killer (NK) cells and cytotoxic T cells, enhancing their ability to recognize and eliminate cancer cells (39–41). Furthermore, IFNs can induce cell cycle arrest and promote apoptosis, thereby inhibiting tumor growth. On the other hand, IFNs can also promote cancer cell survival and resistance to therapies by inducing the expression of immunosuppressive molecules and activating pro-survival signaling pathways (41). The balance between these opposing effects is complex and influenced by the tumor microenvironment, genetic alterations, and individual patient characteristics (41, 42). Understanding the role of IFNs in breast cancer pathogenesis is crucial for developing targeted therapies that harness the beneficial effects of IFNs while minimizing their potentially detrimental consequences (39–42). The unique properties of gold nanoparticles indicate their multiple applicability in several therapeutic applications, such as a carrier in drug and gene delivery and photothermal and photodynamic therapies (22, 43). Furthermore, the applicability of gold nanoparticles has also been tested as antibacterial and antifungal agents. Moreover, the potential of gold nanoparticles as an antioxidant and anticancer agents has to be proved, but further investigations are still required at the clinical level (22, 43–45). Notably, gold nanoparticle AuNPs were also reported to have toxic effects linked to their cellular intake (46). Therefore, safety issues of AuNPs are highly concerned and need to be rectified. Interestingly, experts working on AuNPs have recently addressed this controversial issue, they properly modified the chemical procedure of AuNPs synthesis in various ways, including the conjugation of functional groups or chemical compounds to their surface to normalize their toxicity (22). In this study, we synthesized citrate-conjugated gold nanoparticles (citrate-AuNPs) and the newly synthesized AuNPs were characterized. The UV-Vis spectra of the citrate-AuNPs displayed a surface plasmon resonance peak at a wavelength (λ max) of 524 nm. These surface plasmon resonance results closely matched those of previously reported AuNPs (24, 25, 47), confirming the successful synthesis of the nanoparticles. To assess the stability of the prepared citrate-AuNPs, we measured their zeta potential, particle size, and polydispersity index (PDI). The zeta potential of the citrate-AuNPs was determined to be -32.2 mV, indicating their stability and the presence of a negative charge on their surface. The size of the citrate-AuNPs was calculated to be 28.3 nm, and the low PDI value of 0.435 indicated their uniformity and low dispersity. The parameters obtained in this study for the newly synthesized citrate-AuNPs were consistent with previous investigations (24, 25, 45, 47, 48), validating the reliability of our findings. Additionally, we conducted spectrophotometric analysis to examine the storage stability of the citrate-AuNPs. Various aliquots of the nanoparticles were stored at different temperatures (-80°C, -20°C, 0°C, 4°C, and room temperature) for a period of 2 weeks, and their UV-Vis spectra were compared. Remarkably, all the samples exhibited similar UV-Vis spectra, with the maximum surface plasmon resonance observed at 524 nm. This outcome confirmed the stability of the newly prepared citrate-AuNPs under the tested temperature conditions for a 2-week duration. To further investigate their storage stability, additional citrate-AuNPs samples were stored at 4°C for varying durations ranging from 0 weeks to 6 months. The UV-Vis spectra of the samples remained largely unchanged from 0 to 3 weeks, and after this point, the peak absorbance gradually decreased from 4 weeks to 6 months, albeit not significantly. These findings reaffirmed that the chemically synthesized citrate-AuNPs remained highly stable for up to 3 weeks when stored at 4°C. Based on these investigations, subsequent studies at the cellular level were carried out using citrate-AuNPs stored for a maximum of 3 weeks at 4°C, while the remaining samples were stored at -80°C to preserve their stability.

Triple-negative breast cancer, TNBC was recently reported to develop in 12-25% of all breast cancer patients with a five years survival rate of 8-16% (47, 49, 50). The prevalence rate of TNBC was the same in all age groups of breast cancer patients (51). In contrast, studies also reported that basal TNBC was more common in younger and older women (49). However, the reason behind is still not fully understood. In this study, we used the most commonly studied TNBC cell line, MDA-MB-231, which was established in the 1970s and has been extensively studied as one of the best triple-negative breast cancer cells (51–53). Initially, we evaluated the cytotoxicity of the newly prepared citrate-conjugated gold nanoparticles (citrate-AuNPs) on MDA-MB-231 cancer cells. Our luminescent cell viability assays demonstrated that the percentage of viable MDA-MB-231 cells remained stable up to a concentration of 0.4 µg/ml of citrate-AuNPs for a duration of 72 hours. Based on these results, all subsequent cellular-level studies were conducted using a maximum concentration of 0.4 µg/ml of citrate-AuNPs, and the maximum treatment duration for the cells was set at 72 hours. SOCS1, originally recognized as a tumor suppressor (6, 53), has been found to be overexpressed in various cancer types, including human breast tumors, epidermal tumors, neuronal tumors, and other cancer cells (54–56). Interestingly, in tumor tissues, high expression levels of SOCS1, both at the mRNA and protein levels, have been consistently associated with tumor invasion and thickness. Moreover, its increased expression has been closely linked to disease progression and has been reported to be highest in metastatic tumors (53–58). However, the role of SOCS1 in triple-negative breast cancer (TNBC) patients remains poorly explored and subject to conflicting interpretations. In this study, we investigated the expression of SOCS1 in MDA-MB-231 cells stimulated with 0.1 µg/ml of IFN-γ over varying time intervals ranging from 0.5 to 6.0 hours. The data revealed a significant increase in SOCS1 expression, at both the mRNA and protein levels, after 0.5 hours of IFN-γ treatment in MDA-MB-231 cells. Subsequently, there was a gradual decline in the expression of SOCS1 from 0.5 to 6 hours of IFN-γ treatment. These findings suggest a potential pathogenic role of both IFN-γ and SOCS1 in the progression of TNBC.

MicroRNAs one of the most important classes of nucleic acids, play a key role in regulating pathogenic genes via bindings in 3’UTR of their mRNA sequences. Now miRNAs have been utilized as one of the most valuable therapeutic targets for making strategies against cancer onset (59, 60). In order to investigate the potential regulatory role of miRNAs on the SOCS1 gene in TNBC, we employed a bioinformatic algorithm. The Targetscan algorithm identified a conserved site for hsa-miR155-5p within the 3’UTR of human SOCS1 mRNA. To confirm these computational predictions, we conducted experimental validation using TNBC MDA-MB-231 cells. Stimulation of these cells with IFN-γ at a concentration of 0.1µg/ml for various durations ranging from 0.5 hours to 6 hours led to a gradual increase in hsa-miR155-5p expression. The highest expression level was observed at 6 hours. Conversely, treatment of the MDA-MB-231 cells with the same concentration of IFN-γ for 0.5 hours resulted in a significant upregulation of SOCS1 at both the mRNA and protein levels. However, subsequent treatment durations from 0.5 to 6 hours led to a sequential decrease in the expression of SOCS1 at both the mRNA and protein levels. These findings revealed that the treatment of MDA-MB-231 cells with IFN-γ at a concentration of 0.1 µg/ml for 0.5 hours resulted in minimal hsa-miR155-5p expression and maximum expression of SOCS1. Furthermore, we confirmed the inverse correlation between hsa-miR155-5p and SOCS1 through a luciferase assay, wherein MDA-MB-231 cells were co-transfected with a reporter clone containing the complete 3’UTR of SOCS1 and anti-miR155. The luciferase activity significantly increased in the cells co-transfected with both the SOCS1 3’UTR and anti-miR155 compared to cells transfected with the SOCS1 3’UTR alone. These novel findings provide further confirmation of the interaction between hsa-miR-155-5p and the seed matched sequence located in the 3’UTR of human SOCS1 mRNA.

Upon confirming a negative correlation between hsa-miR155-5p and SOCS1 expression in TNBC MDA-MB-231 cells stimulated with IFN-γ, we investigated the therapeutic potential of citrate-conjugated gold nanoparticles (citrate-AuNPs). Pre-treatment of MDA-MB-231 cells with citrate-AuNPs prior IFN-γ treatment led to a significant dose-dependent inhibition of both SOCS1 mRNA and SOCS1 protein expression. These findings indicate that citrate-AuNPs may be promise source as an anticancer agent against the inflammatory responses reported in TNBC patients. The oncogenic role of miR-155 in breast cancer has been well-established (15), and its involvement in immunoediting cancer and immune cell responses has been discussed in a review by Kalkusova et al. (60). The levels of miR-155 expression in cancer cells and immune cells have been shown to influence the severity of cancer and its response to therapy, making miR-155 an attractive target for cancer diagnostics, prognosis, and treatment (60). In this study, we aimed to investigate whether citrate-AuNPs could suppress the IFN-γ-induced expression of SOCS1 in TNBC and whether this suppression is mediated by hsa-miR155-5p. To test these hypotheses, we conducted luciferase reporter assays in MDA-MB-231 cells. Co-transfection of the cells with a clone of SOCS1 3’UTR and anti-miR-155, followed by treatment with citrate-AuNPs, resulted in a significant increase in relative luciferase activity. However, this increase was completely inhibited when the co-transfected cells were treated with citrate-AuNPs. This suggests that citrate-AuNPs inhibit SOCS1 expression by modulating hsa-miR155-5p expression. Further validating this inhibitory mechanism, we performed transfection experiments with anti-miR155-5p, followed by treatment with citrate-AuNPs. The data revealed that transfection of MDA-MB-231 cells with anti-miR155-5p significantly increased the levels of SOCS1 mRNA and protein. Interestingly, this increase was notably suppressed in a dose-dependent manner by citrate-AuNPs. These findings further support the notion that citrate-AuNPs inhibit SOCS1 expression by modulating hsa-miR155-5p expression. Additionally, we explored the therapeutic effects of citrate-AuNPs in normalizing the upregulated expression of SOCS1 through hsa-miR155-5p. To investigate this, we transfected MDA-MB-231 cells with anti-miR155-3p, followed by co-treatment with citrate-AuNPs and IFN-γ. The data demonstrated that IFN-γ stimulation significantly enhanced the expression of SOCS1 mRNA in anti-miR155-transfected MDA-MB-231 cells. However, treatment with citrate-AuNPs effectively suppressed the IFN-γ-induced expression of SOCS1 mRNA and protein in a dose-dependent manner in MDA-MB-231 cells transfected with anti-miR155-5p. These findings represent a novel and previously unexplored area of research, demonstrating the ability of newly prepared citrate-AuNPs to inhibit the expression of SOCS1 mRNA and protein by modulating hsa-miR155-5p expression.

Nuclear Transcription factor, NFκB, is a potent inflammatory signaling pathway involved in numerous pathogenic processes and the treatment of cancers. Activation of NFκB signaling via its subunit p65 and/or p50 has been involved in tumorigenesis and has now been well considered one of the best targets for cancer therapy (61, 62). This study, demonstrated a significant increase in the levels of NF-κBp65 and NF-κBp50 subunits in the nuclear extract of MDA-MB-231 cells treated with IFN-γ alone for 30 minutes. Similarly, a notable elevation in NF-κB p65 and p50 levels in the nuclear extract was observed in MDA-MB-231 cells transfected with anti-miR155-5p. Interestingly, a marked increase in activated NF-κB p65 and p50 was observed when the transfected MDA-MB-231 cells with anti-miR155-5p were stimulated with IFN-γ. Importantly, this sharp increase in NF-κB p65 and p50 activation was dose-dependently inhibited by treatment with citrate-AuNPs. This reduction in the activation of NF-κB p65 and p50 by citrate-AuNPs suggests that citrate-AuNPs inhibit the activity of NF-κB p65 and p50 by modulating the expression of hsa-miR155-5p. The data indicate that chemically synthesized citrate-AuNPs suppress the expression of SOCS1 by modulating hsa-miR155-5p and deactivating the activity of NF-κB p65/p50 in triple-negative breast cancer cells.

MicroRNA analysis at the cellular level is a valuable tool for studying gene regulation and cellular processes. However, it also has certain limitations when it comes to *in vivo* studies (63). Here are some limitations of miRNA analysis at the cellular level, particularly in the context of *in vivo* studies; *in vivo* studies often involve analyzing miRNAs in complex tissues or whole organisms. Tissues are composed of different cell types, each with its own unique miRNA expression profile. When analyzing miRNAs at the cellular level within tissues, it becomes challenging to distinguish the specific contributions of different cell types to the overall miRNA expression patterns. This tissue heterogeneity can confound the interpretation of cellular-level microRNA analysis; *in vivo* miRNA analysis may lack spatial resolution (64, 65). While it is possible to isolate specific cell populations or use imaging techniques to study miRNA localization, it is often difficult to precisely determine the spatial distribution and activity of miRNAs within tissues (66). This limitation can hinder the understanding of miRNA-mediated cellular communication and signaling processes; in some *in vivo* studies, access to specific target tissues or organs may be limited or invasive. Obtaining sufficient and representative samples for miRNA analysis from these tissues can be challenging, especially in human studies. The availability and quality of samples can vary, potentially introducing biases and limitations in the analysis. MicroRNA expression and activity are highly dynamic and can change rapidly in response to various stimuli or physiological conditions. Analyzing miRNAs at a single time point or over a short duration may not capture the full dynamics of miRNA regulation (67). Longitudinal studies and time-resolved analyses are required to understand the temporal aspects of miRNA-mediated regulatory processes. *In vivo* studies can involve systemic manipulations such as genetic modifications, drug treatments, or disease models. These systemic interventions can affect miRNA expression and function not only in the target tissues but also in other interconnected organs or systems. Understanding the systemic effects of interventions on miRNA regulation requires comprehensive and integrated analyses across different tissues and physiological contexts. Although miRNA expression profiling can provide insights into potential regulatory roles, it does not provide direct information on the functional consequences of miRNA activity. Functional validation experiments are necessary to establish the causal relationship between miRNAs and their target genes, and to determine the specific phenotypic effects resulting from miRNA dysregulation; *in vivo* studies involving miRNA analysis often require animal models or human samples, which can be subject to ethical and practical limitations. Animal models may not perfectly recapitulate human biology, and human studies may be restricted by ethical constraints or limited availability of suitable samples. These considerations can impact the design and feasibility of *in vivo* miRNA studies. Understanding and addressing these limitations are crucial for robust interpretation of miRNA analysis in the context of *in vivo* studies. Integrating multiple experimental approaches, careful experimental design, validation strategies, and cross-species comparisons can help overcome these challenges and improve the reliability and translational relevance of miRNA research at the cellular and *in vivo* levels.

## Conclusions

Newly synthesized gold nanoparticles citrate-AuNPs demonstrated stability and are non-toxic to triple-negative breast cancer cells. Citrate-AuNPs inhibit IFN-γ-induced SOCS1 mRNA and protein expression and deactivate the activity of nuclear transcription factor NF-κB subunits p65 and 50 via negative regulation of microRNA hsa-miR155-5p (**Figure 10**). These novel pharmacological actions of gold nanoparticles citrate-AuNPs on IFN-γ-stimulated TNBC cells provide novel suggestions that AuNPs inhibit IFN-γ induced inflammation in TNBC cells by modulation of expression of microRNAs. These findings have never been reported earlier and demonstrate therapeutic value for designing novel therapies for treating patients with triple-negative breast cancer.

## Data availability statement

The original contributions presented in the study are included in the article/supplementary materials, further inquiries can be directed to the corresponding author/s.

## Ethics statement

Ethical approval was not required for the studies on humans in accordance with the local legislation and institutional requirements because only commercially available established cell lines were used.

## Author contributions

AF, ZR, and AA were responsible for writing the manuscript, conceiving the experimental study design, and analyzing the data. ZR and AF performed the experiments, produced the figures, and performed the statistical analysis. NR critically analyzed and edited the manuscript. All authors contributed to the article and approved the submitted version.
